# Multifocal electroretinogram and Optical Coherence tomography spectral-domain in arc welding macular injury: a case report

**DOI:** 10.1186/1471-2415-11-40

**Published:** 2011-12-30

**Authors:** Mauro Cellini, Roberto Gattegna, Pier Giorgio Toschi, Ernesto Strobbe, Emilio C Campos

**Affiliations:** 1 Department of Specialistic Surgery and Anesthesiology Science, University Ophthalmology Unit, S. Orsola Malpighi-Hospital, Pelagio Palagi 9, Bologna, 40138, Italy

## Abstract

**Background:**

the purpose of this study was to report a binocular photic retinal injury induced by plasma arc welding and the follow-up after treatment with vitamin supplements for a month. In our study, we used different diagnostic tools such as fluorescein angiography (FA), optical coherence tomography (OCT) and multifocal electroretinogram (mfERG).

**Case presentation:**

in the first visit after five days from arc welding injury in the left eye (LE) the visual acuity was 0.9 and 1.0 in the right eye (RE). FA was normal in both eyes. OCT in the left eye showed normal profile and normal reflectivity and one month later, a hyperreflectivity appeared in the external limiting membrane (ELM). The mfERG signal in the LE was 102.30 nV/deg2 five days after the injury and 112.62 nV/deg2 after one month and in the RE respectively 142.70 nV/deg2 and 159.46 nV/deg2.

**Conclusions:**

in cases of retinal photo injury it is important for the ophthalmologist to evaluate tests such as OCT and the mfERG in the diagnosis and follow-up of the patient because the recovery of visual acuity cannot exclude the persistence of phototoxic damage charged to the complex inner-outer segment of photoreceptors.

## Background

The light emitted during the use of welding tools is known to be a source of injuries to various structures of the eye. The most frequent damage is actinic or photoelectric keratoconjunctivitis, which affects the ocular surface [1], but in some cases retinal structures may also be involved.

Each instrument used to weld produces, depending on the technology used, a specific type of optical radiation. Despite this, metal arc welding, tungsten arc welding and gas arc welding mainly generate ultraviolet spectrum waves [2,3].

Welding techniques in recent years have gradually improved, and plasma welding has recently been gaining more widespread use because it allows for faster and more accurate welding compared to before.

However, this increasingly popular technique produces a large amount of electromagnetic waves, resulting in a high operating temperature, with an increased risk of retinal damage.

Regarding the damage to the posterior structures of the eye, we know that this is caused by radiation with a wavelength between 400 and 1400 mM, as wavelengths between 100 and 400 μm are absorbed by the cornea and lens, and in particular those between 400 and 500 μm [4].

Acute phototoxic damage is sustained by retinal pigment epithelium (RPE) depigmentation and a swelling of the outer retinal layers, and secondly, the damage is transmitted to the inner layers of the retina [5].

Cases of macular degeneration due to welding described in the literature are quite rare, especially with regards to the study of retinal lesions with optical coherence tomography (OCT) and functional damage with multifocal electroretinogram (mfERG).

In this article, we report the case of a bilateral maculopathy induced by plasma arc welding, studied with OCT and mfERG.

## Case presentation

A 26 year-old male subject visited the emergency eye clinic for the appearance of persistent blurred vision in his left eye arising 4-5 days previously. His anamnesis reports having assisted with work on plasma arc welding, about a week before the visit, without the use of protective lenses.

The visual acuity of the left eye was 0.9 and 1.0 in the right eye. The biomicroscopic examination of the anterior segment of the diopters of both eyes appeared unharmed and normally transparent. The ophthalmoscopic examination detected an abnormal macular reflex of the left eye, which was characterized by a round yellow lesion in the centre of the fovea.

The right eye, optic disc, macula and vessels appeared normal. The patient underwent FA and OCT with Spectralis HRA-OCT (Heidelberg Engineering, Heidelberg, Germany) and mfERG using electroretinography RetinaxPlus (CSO, Florence, Italy) according to ISCEV guidelines [6].

**Figure 1:**
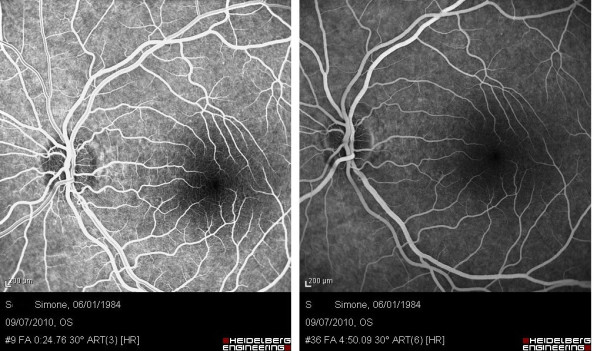
**Arteriovenous angiographic time (left) and later stages of angiography (right) of the left eye 5 days after macular photo injury**.

**Figure 2:**
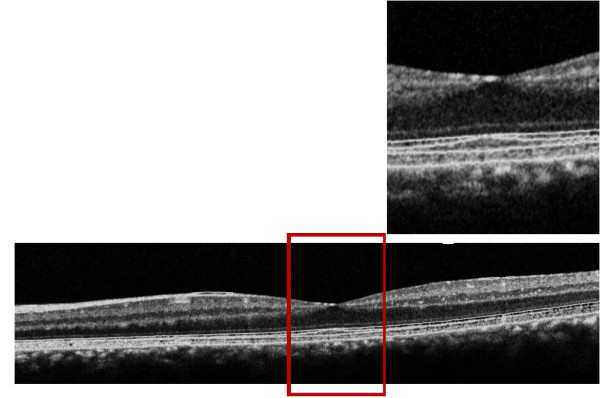
**OCT of the left eye 5 days after macular photo injury OCT showed normal profile and normal reflectivity**.

In the left eye, FA showed no retinal changes in chorioretinal circulation (Figure 1) and OCT showed a normal profile and normal reflectivity (Figure 2).

**Figure 3:**
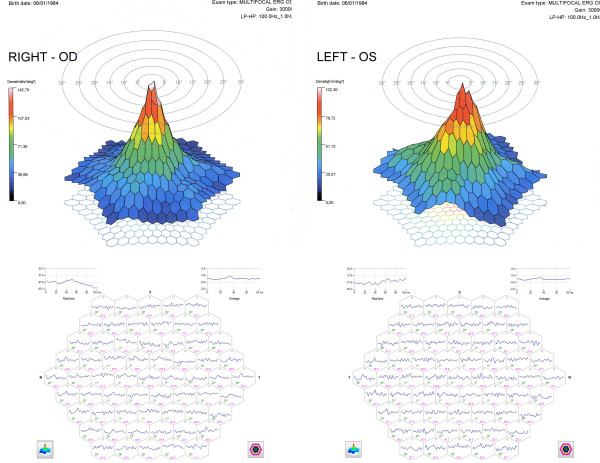
**The 3D mfERG (top) and the 63 mfERG responses (first-order kernel) (bottom) of both eyes 5 days after macular photo injury**.

Finally, mfERG showed a change in the signal on the central photoreceptors of the central 2° of the left eye with a value of 102.30 nV/deg2 and an alteration of the signal only on the central photoreceptors of 1° of the right eye, with a load value average of 142.70 nV/deg2 (Figure 3).

Treatment with vitamin supplements, mainly lutein, astaxanthin, zeaxanthin, folic acid, selenium, vitamin C, zinc and ginkgo biloba was prescribed for a month.

During the follow-up visit, two weeks after the end of the treatment, the visual acuity was 1.0 in the left eye and the ophthalmoscopic macular alteration, reported previously, had disappeared.

**Figure 4:**
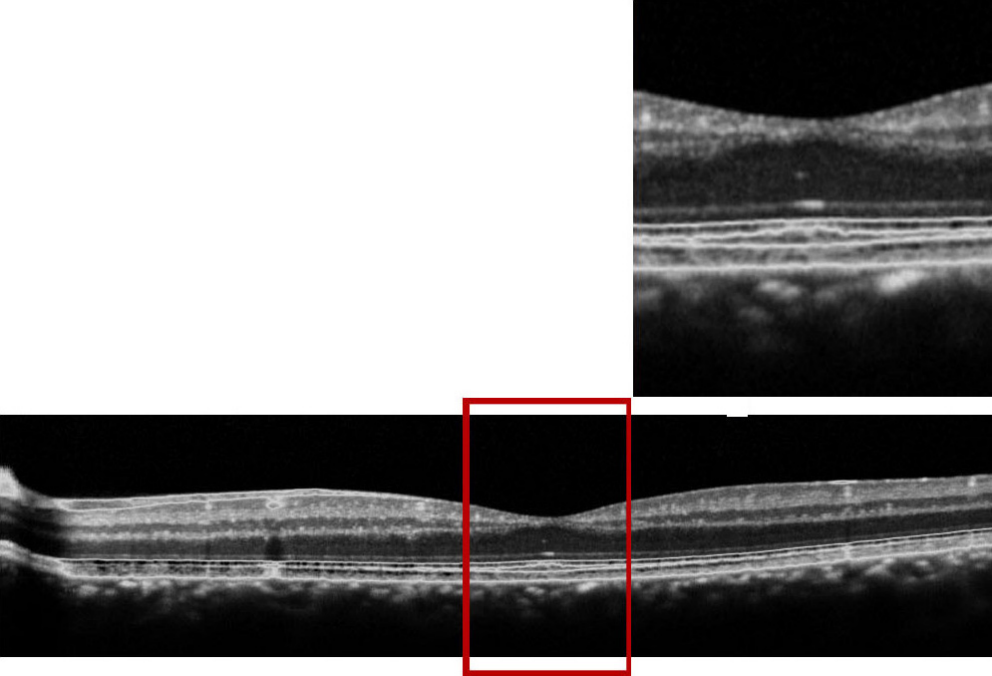
**OCT of the left eye one month after macular photo injury where we see a hyperreflectivty in the external limiting membrane**.

The patient underwent an OCT and mfERG check-up and refused to undergo the FA again. The OCT showed a hyperreflectivity of the ELM (Figure 4).

**Figure 5:**
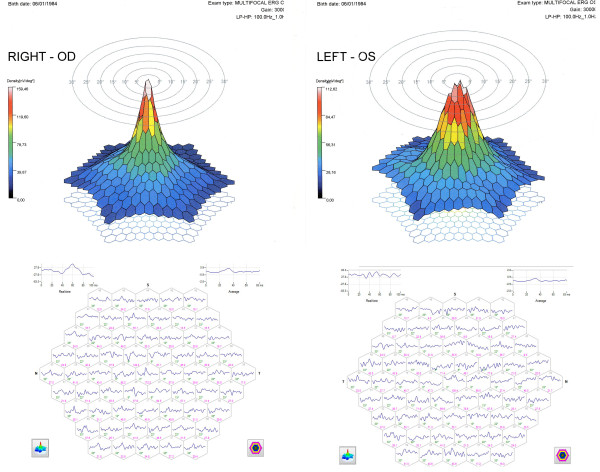
**The 3D mfERG (top) and the 63 mfERG responses (first-order kernel) (bottom) of both eyes one month after macular photo injury**.

The mfERG showed an improvement of the track in the central 2° in both eyes with an average value of 159.46 nV/deg2 in the right eye and 112.62 nV/deg2 in the left eye where the beheading of the signal in the 2° persisted (Figure 5).

## Discussion

Photo injuries arising from the use of arc welding are quite rare, and the first case was described by Terrier in 1902. The main cause of the appearance of macular degeneration is due to the failure to use proper eye protection [7].

Electrical systems in place for welding emit electromagnetic waves at high temperature and frequency ranges; from ultraviolet to the blue spectrum, however, all radiation can damage the ocular structures.

Today the most commonly welding techniques that use the ionized plasma of noble gases can cause retinal damage.

These gases are brought to temperatures so high that single molecules are broken down into atoms and then into electrons and protons, which is the so-called fourth phase of matter or the plasma phase. The plasma results from a marked rise in temperature that can reach values between 10,000 and 30,000°C, emitting light radiation harmful to retinal structures.

Phototoxic retinal damage appears to be multifactorial and involves several mechanisms of action depending on the chromophore involved in the bright damage.

The visual pigments, rhodopsin in particular, are among the main chromophores responsible for such damage, and lead to the alteration of cellular function and cytotoxicity.

The mechanism of action of rhodopsin mainly occurs in two ways: the first due to a prolonged activation of rhodopsin as meta-rhodopsin, which leads to a reduction of the concentration of intracellular calcium, initiating apoptosis, and the second through the issuance of phototoxic substances such as retinal [8]. Histological examination immediately following light exposure reveals that photoreceptor cell damage begins at the apex of the photoreceptor outer segment and advances over time to include the entire outer segment [9-12].

However, given the large number of phagosomes detected in the RPE, the photodamaged outer segment discs are digested, leading to a general decrease in the length of the photoreceptor outer segment [13].

The cascade of photochemical reactions may also release free radicals, superoxide anions and hydrogen peroxide, which react with the tissue and cell membranes to form aldehydes. If these substances are not readily degraded, the damage to the photoreceptor can be permanent.

What makes the case interesting is that the patient was not a welder and had monocular symptoms having seen only once a plasma welding process. The FA was normal but with OCT we found changes in the reflectivity of ELM and mfERG showed a reduction in amplitude in the central 2° in both eyes.

The negative FA did not help to address our initial diagnosis, also because of frequent negative retinal angiography in cases of photo trauma [14]. Therefore, it was important to perform OCT [15-17] and mfERG [18].

In our case the spectral-domain OCT showed a hyperreflectivity area in the ELM. This particular aspect was not detected with previous time-domain OCT technique [7]. In cases of photo injury assessed with spectral-domain technique a normal ELM was found [19], together with frequent hyporeflective space between the outer and inner hyperreflective layers and RPE-choriocapillaris complex [17]. This last OCT finding is similar to cases of solar retinopathy [20] whereas phototoxic effect is associated with a direct thermal damage of the photoreceptor outer segment-RPE complex. The mfERG showed a reduction in the amplitude in the central 2° in both eyes; this reduction has been improving over time as confirmed by control mfERG made a month later, confirming the importance of this exam in the diagnosis and in the follow-up of retinal photo injury [18,20].

As for the asymmetrical involvement, this was likely due to the patient's positioning with respect to the welding tool, rather than a difference in the ocular structures in terms of sensitivity to photo damage.

With regard to the treatment of this disease, the data seem to be discordant regarding the use of corticosteroids [21-23]. The use of vitamin A and aspirin appears to reduce the risk of phototoxic damage to the retina [24], similar to the use of antioxidants such as vitamins B, C and E and ginkgo biloba [25,26].

In our case after treatment with antioxidants, we observed a resolution of visual symptoms with improvement of the mfERG values but the persistence of phototoxic damage charged to the external limiting membrane found with OCT.

## Conclusions

In conclusion, we believe that great attention should be placed during the welding process, including staff not directly involved but present in the workplace, who should wear appropriate protective eyewear. Finally, we believe that in cases of retinal photo trauma, the implementation of OCT and particularly mfERG is of great importance to assess over time the degree of recovery of retinal function.

## Competing interests

The authors declare that they have no competing interests.

## Authors' contributions

MC recruited the patient from the Ophthalmology First Aid of the S. Orsola-Malpighi Hospital and evaluated mfERG. RG and PGT drafted the manuscript and reviewed the literature. ES and ECC evaluated retinal angiography and OCT. All authors read and approved the final manuscript.

## Consent

Mauro Cellini, MD that examined the patient, received the informed written consent from the patient for publication of the manuscript and any accompanying images.

## Pre-publication history

The pre-publication history for this paper can be accessed here:

http://www.biomedcentral.com/1471-2415/11/40/prepub

## References

[B1] Rieke FE Arc flash conjunctivitis J Am Med Ass 1943 122 734 36 10.1001/jama.1943.02840280018005

[B2] Tenkate TD Optical radiation hazards of welding arcs Rev Environ Health 1998 13 131 46 10.1515/REVEH.1998.13.3.131 9842654

[B3] Okuno T Saito H Ojima J Evaluation of blue-light hazards from various light sources Dev Ophthalmol 2002 35 104 12 12061267 10.1159/000060814

[B4] Brittain GP Retinal burns caused by exposure to MIG-welding arcs: report of two cases Br J Ophthalmol 1988 72 570 5 10.1136/bjo.72.8.570 3415950 PMC1041530

[B5] Mainster MA Turner PL Ryan SJ, Hinton DR, Schachat AP, Wilkinson P Retinal injuries from light: Mechanisms, Hazards and Prevention Retina 2006 2 4 Mosby Elsevier Publishers

[B6] Hood DC Bach M Brigell M Keating D Kondo M Lyons JS Palmowski-Wolfe AM ISCEV guidelines for clinical multifocal electroretinography Doc Ophthalmol 2008 2007 116 1 11 10.1007/s10633-007-9089-2 PMC2235911 17972125

[B7] Choi SW Chun KI Lee SJ Rah SH A case of photic retinal injury associated with exposure to plasma arc welding Korean J Ophthalmol 2006 20 250 3 10.3341/kjo.2006.20.4.250 17302214 PMC2908862

[B8] Maier R Heilig P Winker R Neudorfer B Hoeranter R Ruediger H Welder's maculopathy? Int Arch Occup Environ Health 2005 78 681 5 10.1007/s00420-005-0013-3 16021465

[B9] Boulton M Rózanowska M Rózanowski B Retinal photodamage J Photochem Photobiol 2001 64 144 61 10.1016/S1011-1344(01)00227-5 11744401

[B10] Bush RA Remé CE Malnoë A Light damage in the rat retina: the effect of dietary deprivation of N-3 fatty acids on acute structural alterations Exp Eye Res 1991 53 741 52 10.1016/0014-4835(91)90109-R 1838336

[B11] Vaughan DK Nemke JL Fliesler SJ Darrow RM Organisciak DT Evidence for a circadian rhythm of susceptibility to retinal light damage Photochem Photobiol 2002 75 547 53 10.1562/0031-8655(2002)075<0547:EFACRO>2.0.CO;2 12017483

[B12] Remé CE The dark side of light: rhodopsin and the silent death of vision. The Proctor Lecture Invest Ophthalmo Vis Sci 2005 46 2671 82 10.1167/iovs.04-1094 16043837

[B13] Organisciak DT Vaughan DK Retinal light damage: mechanisms and protection Prog Retin Eye Res 2010 29 113 34 10.1016/j.preteyeres.2009.11.004 19951742 PMC2831109

[B14] Freeman J Gombos GM Fluorescein fundus angiographyin self-induced solar retinopathy Can J Ophthalmol 1971 6 124 7 5148217

[B15] Mainster MA Boulton M Albert DM, Miller JW, Blodi BA, Azar DT, et al Photic retinopathy Albert and Jakobiec's Principles and Practice of Ophthalmology 2008 2 3 Edinburgh: Saunders 2195 2051374874

[B16] Huang SJ Gross NE Costa DL Yannuzzi LA Optical coherence tomography findings in photic maculopathy Retina 2003 23 863 6 10.1097/00006982-200312000-00020 14707841

[B17] Vedantham V Optical coherence tomography findings in a case of chronic welder's maculopathy Eye 2006 20 269 71 10.1038/sj.eye.6701846 15776008

[B18] Denk PO Kretschmann U Gonzalez J Gelisken F Knorr M Phototoxic maculopathy after arc welding: value of multifocal ERG Klin Monatsbl Augenheilkd 1997 211 207 10 10.1055/s-2008-1035122 9445902

[B19] Pilli S Ogoti M Kalluri V Fourier-domain optical coherence tomography findings in welder's maculopathy Ophthalmic Surg Lasers Imaging 2010 1 5 10.3928/15428877-20100215-93 20337280

[B20] Stangos AN Petropoulos IK Pournaras JA Zaninetti M Borruat FX Pournaras CJ Optical coherence tomography and multifocal electroretinogram findings in chronic solar retinopathy Am J Ophthalmol 2007 144 131 4 10.1016/j.ajo.2007.03.003 17601436

[B21] Cellini M Profazio V Fantaguzzi P Barbaresi E Longanesi L Caramazza R Photic maculopathy by arc welding. A case report Int Ophthalmol 1987 10 157 9 3596908 10.1007/BF00139342

[B22] Arend O Aral H Reim M Wenzel M Welder's maculopathy despite using protective lenses Retina 1996 16 257 9 10.1097/00006982-199616030-00014 8789868

[B23] Vedantham V Correspondence Retina 2005 25 1122 author reply p.1122 16340558 10.1097/00006982-200512000-00035

[B24] Shahriari HA Salari AM Preventive effects of Vitamin A and Aspirin on the UV light-induced retinopathy in an animal model Zahedan University of Medical Sciences, 98134 Zahedan, Iran

[B25] Rhone M Basu A Phytochemicals and age-related eye diseases Nutr Rev 2008 66 465 72 10.1111/j.1753-4887.2008.00078.x 18667008

[B26] Ritch R Natural compounds: evidence for a protective role in eye disease Can J Ophthalmol 2007 42 425 38 10.3129/i07-044 17508040

